# Influence of the built environment of Nanjing’s Urban Community on the leisure physical activity of the elderly: an empirical study

**DOI:** 10.1186/s12889-019-7643-y

**Published:** 2019-11-06

**Authors:** Zhi-jian Wu, Yanliqing Song, Hou-lei Wang, Fan Zhang, Fang-hui Li, Zhu-ying Wang

**Affiliations:** 1 School of Sport Sciences, Nanjing Normal University, No. 1 Wenyuan Road Qixia District, Nanjing, 210046 China; 2 Physical Education Department, Nanjing University of Posts and Telecommunications, Nanjing, China; 3 School of Police Skills and Tactics, Nanjing Forest Police College, Nanjing, China

**Keywords:** Elderly, Recreational physical activity, Demographic factors, Built environment factors, Empirical research

## Abstract

**Background:**

Urbanization and aging are global phenomena that offer unique challenges in different countries. A supportive environment plays an important role in addressing the issues of health behavioral change and health promotion (e.g., prevent chronic illnesses, promote mental health) among older adults. With the development of the socio-ecological theoretical model, studies on the impact of supportive environments on physical activity have become popular in the public health field in the EU and US. Meanwhile, very few Chinese studies have examined the relationship between built environment features and older adults’ physical activity at the ecological level. The purpose of the study is to investigate how the factors part of the built environment of Nanjing’s communities also influence leisure time physical activity among the elderly.

**Methods:**

Using a socio-ecological model as a theoretical framework, we conducted a cross-sectional study of 399 elderly people from 19 communities in Nanjing, China, using a one-on-one questionnaire to collect data, including participants’ perceived built environment and self-reported physical activity. A multivariate linear regression method was used to analyze the factors influencing their recreational physical activity.

**Results:**

This study found that compared to older people with low average monthly income, the recreational physical activity of the elderly with average monthly incomes between 1001 and 2000 ¥ (β = 23.31, *p* < 0.001) and 2001 ¥ or more (β = 21.15, p < 0.001) are significantly higher. After controlling for individual covariates, street connectivity (β = 7.34, *p* = 0.030) and street pavement slope (β = − 7.72, *p* = 0.020), we found that two out of ten built environment factors indicators influence their physical activity. The importance of each influencing factor ranked from highest to lowest are monthly average income, street pavement slope, and street connectivity. Other factors were not significantly related to recreational physical activity by the elderly.

**Conclusions:**

Older adults with a high income were more likely to participate in recreational physical activity than those with a low income. In order to positively impact physical activity in older adults and ultimately improve health, policymakers and urban planners need to ensure that street connectivity and street pavement slope are factored into the design and development of the urban environment.

## Background

The growing aging population and the resulting socioeconomic pressure constitute a serious problem facing Chinese society and threaten to continue to do so [1]. It is estimated that the population of China over the age of 60 will increase from 15.500% (212 million) in 2014 to 28.000% (402 million) in 2040; moreover, the number of people over the age of 80 will increase to 90.4 million in 2050, giving China the largest elderly population in the world [2]. As the population ages, age-related diseases such as ischemic heart disease, cancer, stroke, arthritis, Alzheimer’s disease, and other chronic (non-infectious) diseases will rise among the population [3, 4]. Studies have shown that regular participation in recreational physical activity has many benefits for physical health, including delaying aging, reducing the incidence of non-infectious diseases, and improving quality of life [5, 6]. *Physical activity* refers to any body movement that involves energy expenditure caused by skeletal muscle contraction and consists mainly of four types: work-related, family-related, traffic-related, and leisure-time physical activity [7]. The World Health Organization (WHO) recommends that middle-aged and elderly people participate in at least 150 min of moderate-intensity physical activity every week [8]. However, despite the obvious benefits of physical activity, the percentage of the population that achieves the recommended amount of exercise decreases with age. According to the results of the global health survey developed by the WHO in 2015, approximately one-third of individuals between 70 and 79 years of age and one-half of those 80 and older do not reach the recommended physical activity standards [9]. Therefore, it is critical to investigate the influencing factors that promote physical activity among the elderly.

The socio-ecological model indicates that human health behavior is influenced by factors such as individual characteristics, interpersonal relationships, environment, and policy [10]. Therefore, individual characteristics and built environment are important factors affecting the physical activity of the elderly [11]. Complex and dynamic interrelations between individual, social, and environmental factors shape physical activity and behavior [12]. The impact of supportive environments on physical activity is a popular topic among public health researchers in Europe and the United States [13]. Socio-ecological models posit that understanding the impact of built environmental attributes on older adults’ physical activity is particularly pertinent, as their diminished physical capacity makes them more vulnerable to the detrimental effects of physically challenging environments (e.g., inclines, residential density, mixed land use, destination accessibility, aesthetics, street connectivity, parks and open space) on physical activity [14]. However, a previous review of the built environment and older adults’ physical activity identified inconsistent correlates [15]. Furthermore, Van Cauwenber et al. systematically analyzed the correlation between physical activity among the elderly and the built environment; the results were heterogeneous (there were no correlations or opposite relationships) [16]. A study by Hanibuchi found that population density and parks or greenspaces were positively correlated with the frequency of physical activity of Japanese elderly people [17]. Another study by Smith et al., comparing the frequency of park and greenspace use by elderly men with that of elderly women, found that elderly women used parks and greenspaces at a lower frequency than men did, and parks and greenspaces only promoted recreational physical activity in elderly men [18]. In terms of traffic, Sallies et al. suggest that strengthening traffic safety can help promote outdoor walking by elderly people [19], whereas McGinn et al. argue that traffic safety is irrelevant to both traffic-related and recreational physical activity [20]. Although studies in the United States, Europe, and Australia agree that the built environment is closely related to physical activity, their findings do not apply to China, which features different urban forms, lifestyles, and cultures. Therefore, more studies in this field that focus on the Chinese context are required. Moreover, research on the relationship between the built environment and physical activity is needed to provide improvement strategies for building environment-friendly communities (e.g., neighborhoods with high levels of safety from traffic and crime, high levels of mixed land use, high-quality public open spaces and parks) that will improve leisure time physical activity of the elderly.

In light of this observation, this study employs a socio-ecological theoretical model as a guide for investigating the factors that influence recreational physical activity among the elderly in China. In doing so, it provides a reference for follow-up exploratory studies in this field in China.

## Methods

The study was conducted in Nanjing, a national central city in eastern Jiangsu Province. In 2017, the population of Nanjing reached 8.335 million residents, of whom slightly more than one-fifth (20.850%) were over 60 years of age. Nanjing’s elderly population is characterized by high cardinality, proportion, growth, age, and support. As a national central city in China, Nanjing is representative of Chinese cities to some extent. The overall goal of this study is to understand the impact of the built environment of communities and the related demographic factors upon recreational physical activity among the elderly by answering two research questions: (a) What is the relationship between recreational physical activity among the elderly and the characteristics of the built environment of communities? and (b) What is the relationship between recreational physical activity among the elderly and demographic factors?

### Data sources and collection

A multistage stratified sampling method was used from May to August 2016. Nineteen communities from the four administrative districts including Qinhuai District, Xuanwu District, and Baixia District of the main city and the suburban Qixia District were selected based on the characteristics of Nanjing’s geographical location (Downtown, Old Town, Suburbs). A cross-sectional survey of random samples of elderly people was conducted in the 19 communities. The study protocol and informed consent procedure were approved by the Ethics Committee in Research of Human Subjects at Nanjing Normal University.

### Sampling

The following inclusion criteria were used in screening participants: Participants should (a) be over 60 years of age at the time of the survey, (b) be residents of the selected communities, (c) have lived in the area for at least 6 months, and (d) have no cognitive impairment but understand that recreational physical activity entails walking to parks, open spaces, and other sites. Thirty elderly people from each community were recruited as subjects, 15 men and 15 women, using face-to-face survey methods to assist them in completing the questionnaire. Eventually, 450 elderly people completed the survey, 51 invalid questionnaires were eliminated, and 399 valid questionnaires were recovered for a response rate of 88.7% [21]. There is a gender imbalance in the sample ultimately used: 177 men and 222 women.

### Study measures and variables

All collected data include basic information about the elderly participant (gender, age, highest educational level, average monthly income, chronic disease, self-assessment health, and living situation), intensity and time of physical activity, and perceived built environment.

#### Access to facilities

The facility access scale was adapted from the widely used Neighborhood Environment Walkability Scale (NEWS) developed by Sallis et al. (α = 0.777) [22]. Test–retest reliability and several indicators of validity have been reported for NEWS in multiple studies [23, 24]. Details about the questionnaire, including response formats and scoring, are available.^1^ Higher scores are presumed to indicate more favorable environments for leisure time physical activity. Participants were asked to rate their level of agreement (strongly agree, slightly agree, neutral, slightly disagree, strongly disagree) on a 5-point Likert-type scale (5 = “strongly agree” and 1 = “strongly disagree”) to the following statements: (a) It is easy to reach exercise facilities (e.g., parks, squares) from home; (b) It is easy to reach commercial facilities (e.g., pharmacies, supermarkets) from home; (c) It is easy to reach service facilities (e.g., hospitals, post offices) from home; (d) There are many entertainment facilities (e.g., cardrooms) near home; (e) It is easy to reach public transportation stations (e.g., bus, subway) from home.

#### Community safety

The community safety scale was adapted from the NEWS and is reliable (α = 0.634) [22–24]. Participants were asked about their level of agreement (strongly agree, slightly agree, neutral, slightly disagree, strongly disagree), on a 5-point Likert-type scale, (1 = “strongly agree” and 5 = “strongly disagree”) to the following statements: (a) The crime rate near my home makes it unsafe to walk during the day; (b) There are stray animals near my home; and (c) There are no rest seats or bathrooms without handrails in the community.

#### Aesthetics

In addition to the objectively measured community environment variables (e.g., residential density), an aesthetics subscale was also included in the NEWS. The subscale has good validity and reliability (α = 0.687) [22–24]. The aesthetic subscale comprised three items: neighborhood vegetation, cleanliness, and cultural landscape [25]. Participants were asked about their level of agreement (strongly agree, slightly agree, neutral, slightly disagree, strongly disagree) on a 5-point Likert-type scale (5 = “strongly agree” and 1 = “strongly disagree”) [26].

#### Residential suitability

Participants completed the NEWS, which assessed residential density, mixed land use, and building type. The scale has good validity and reliability (α = 0.643) [22–24]. The extent to which the elderly individuals perceived each variable (strongly agree, slightly agree, neutral, slightly disagree, strongly disagree) was scored using a 5-point Likert-type scale (5 = “strongly agree” and 1 = “strongly disagree”) [26].

#### Traffic safety

The traffic safety scale was adapted from the NEWS and consisted of four questions concerning street pavement flatness, traffic volume, traffic safety, and street connectivity [22]. Test–retest reliability and several indicators of validity have been reported for the NEWS in multiple studies (α = 0.797) [23, 24]. The extent to which the elderly individuals perceived each variable (strongly agree, slightly agree, neutral, slightly disagree, strongly disagree) was scored using a 5-point Likert-type scale (5 = “strongly agree” and 1 = “strongly disagree”) [26].

#### Physical activity

The Physical Activity Scale for the Elderly (PASE) questionnaire was developed in the United States in 1993 by Washburn et al. to investigate physical activity of the elderly population [26]. The current study extracted questions related to recreational physical activity from PASE questionnaires with good reliability and validity to employ as a measurement tool for physical activity. The questionnaire was used to survey the number of days of recreational physical activity, type of exercise, and time spent exercising for each instance in the 7 days preceding the survey. The intensity and type of exercise in this questionnaire primarily included walking; mild-intensity (slight warmth and no sweating), moderate-intensity (warmth and light sweating), and high-intensity physical activity (heavy sweating); and strength training. Based on the collected information, recreational physical activity was scored using frequency and item category weighting: the total score of recreational physical activity = frequency of activity (days per week) × activity time (hours per day)÷7 days×weighted index for each activity. These component scores, representing our most refined estimates of the underlying physical activity construct, were then regressed on responses to the questionnaire to derive the optimal item weights for the PASE. Item weights for the PASE are as follows: 20 for outdoor walking, 21 for mild activity, 23 for moderate activity, 23 for strenuous activity, 30 for muscle endurance activity, 36 for lawn work or yard care, and 20 for outdoor gardening. The total PASE score in this sample of older individuals ranged from 0 to 360; the higher the score, the greater the amount of physical activity [27].

### Statistical analysis

All analyses were performed using STATA 12.0 and multivariate linear regression models were used to analyze the relationship between built environmental factors and demographic factors and recreational physical activity among the elderly [28]. The reliability of the content was first evaluated according to Cronbach’s α coefficient (α = 0.869). Next, Spearman’s correlation analysis was used to analyze the correlated built environment factors. Factor analysis was used to reduce the dimensionality of built environment factors (e.g., community safety, cultural landscape, diversity of mixed land use, residential density, geographic location, street pavement slope, traffic volume, traffic safety, and street connectivity). The built environment factors were extracted by meta-analysis and then systematic review and NEWS; next, principal component factors were extracted by factor analysis.

Finally, linear regression models were used to study the effects of built environmental factors and demographic factors on recreational physical activity among the elderly. Model 1 is a univariate model enabling the investigation of the relationship between demographic factors and recreational physical activity. Model 2 is a univariate model to investigate the relationship between built environment factors and recreational physical activity. Model 3 analyzes the effects of built environmental factors on recreational physical activity with demographic factors as a covariate.

## Results

### General status and environmental characteristics

The demographic characteristics of the 399 respondents are shown in Table 1. Overall, 222 of the older adults who participated were women (55.6%); more than half of the participants (61.9%) were between 60 and 70 years old. Only 8.8% graduated from university. More than half lived alone at the time of this study, and 61.4% had a monthly income of more than 2001 ¥. More than 60.2% reported having chronic disease, and 48.9% reported good self-rated health. The PASE score was 68.9 ± 40.3. The built environment factors for the 399 respondents are shown in Table 2.

**Table 1:** List of basic information of respondents (*n* = 399)

Variable	Frequency	Percentage/%	Variable	Frequency	Percentage/%
Gender			Average monthly income(¥)		
Male	177	44.4	Below 1000	104	26.1
Female	222	55.6	1001–2000	50	12.5
Age			2001or more	245	61.4
60–65 years	141	35.3	Self-evaluation health		
66–70 years	106	26.6	Poor	29	7.3
71–75 years	72	18.0	Average	175	43.8
76 or more	80	20.1	Good	195	48.9
Highest level of education			Living situation		
Elementary school	152	38.1	Alone	228	57.1
Junior high school	127	31.8	Together	171	42.9
Senior high school	85	21.3	Chronic disease		
University	35	8.8	Yes	240	60.2
PASE score	68.9 ± 40.3		No	159	39.8

**Table 2:** List of built environment factors (n = 399)

Variable	M ± SD	Variable	M ± SD
Traffic safety	4.36 ± 0.76	Geographical location	3.91 ± 0.87
Street pavement slope	4.43 ± 0.73	Building Type	4.17 ± 0.80
Street connectivity	4.16 ± 0.79	Mixed land use	4.12 ± 0.85
Sitting facilities	4.38 ± 0.74	Natural landscape	4.38 ± 0.75
Access to services	4.35 ± 0.83	Aesthetics	4.12 ± 0.78
Access to commercial	4.32 ± 0.83	Environmental sanitation	4.60 ± 0.68
Access to fitness	4.42 ± 0.77	Traffic volume	4.33 ± 0.76
Access to Entertainment	4.33 ± 0.83	Community security	4.61 ± 0.71
Traffic site	4.27 ± 0.87	Active environment	4.56 ± 0.69
Residential density	4.04 ± 0.81		

### Factor analysis of built environment factors

The correlation analysis results are shown in Table 1. To validate the rationality of the built environment factors, a confirmatory factor analysis was performed for each. The results of confirmatory factor analysis of perceived built environmental factors. KMO = 0.869, and statistical significance was determined; this indicated that factor analysis could be performed on the built environmental factors. The results of factor analysis (Table 3) show that five principal components can explain 58.91% of the total variables, and five principal components (1, 2, 3, 4, 5) can generally reflect the status of the built environment factors. From the size of the factor loadings of each variable in the first five principal components shown in Table 4, factors less than 0.3 are not shown in the table, and between one and three factors were selected as the representatives of each principal component to exemplify the characteristic indicator of each class of factors. Principal component 1 comprised traffic safety, street pavement slope, and street connectivity. Principal component 2 comprised service facility and access to exercise facilities. Principal component 3 comprised geographical location and residential density. Principal component 4 comprised natural landscape and cultural landscape. Principal component 5 comprised community safety. Principal components 1 through 5 respectively represent traffic accessibility, access to destinations, residential suitability, residential aesthetics, and living environment and facility safety.

**Table 3:** Summary of eigenvalues and contribution rates obtained by principal component analysis

	Start feature value			extract square sum load			cycle square sum load		
	total	Variance %	Accumulate %	total	Variance %	Accumulate %	total	Variance %	Accumulate %
1	5.78	30.40	30.40	5.78	30.40	30.40	2.60	13.69	13.69
2	2.05	10.77	41.17	2.05	10.77	41.17	2.56	13.46	27.15
3	1.29	6.77	47.94	1.29	6.77	47.94	2.14	11.28	38.43
4	1.12	5.88	53.82	1.12	5.88	53.82	2.08	10.96	49.40
5	0.97	5.09	58.91	0.97	5.09	58.91	1.81	9.51	58.91
6	0.94	4.97	63.89						
7	0.90	4.73	68.62						
8	0.70	3.69	72.31						
9	0.68	3.59	75.90						
10	0.58	3.06	78.96						
11	0.56	2.95	81.91						
12	0.56	2.92	84.83						
13	0.53	2.80	87.63						
14	0.47	2.50	90.13						
15	0.46	2.44	92.57						
16	0.42	2.20	94.77						
17	0.35	1.85	96.62						
18	0.35	1.82	98.44						
19	0.30	1.56	100.00						

Extraction method: principal component analysis

**Table 4:** List of variance maximal rotation factor load matrix

	Principal component 1	Principal component 2	Principal component 3	Principal component 4	Principal component5
Traffic safety	0.74				
Street pavement slope	0.70		0.31		
Street connectivity	0.68				
Sitting facilities	0.68				0.35
Access to services		0.80			
Access to commercial		0.70			0.32
Access to fitness		0.70			
Access to Entertainment		0.61		0.46	
Traffic site		0.57	0.33		0.41
Residential density			0.67		
Geographical location	0.31		0.64		
Building Type			0.61		
Mixed land use	0.30		0.43		
Natural landscape				0.72	0.30
Aesthetics				0.70	
Environmental sanitation				0.50	0.37
Traffic volume	0.35		0.34	0.48	
Community security					0.78
Active environment				0.30	0.69

Extraction method: principal component analysis

### Factors influencing recreational physical activity among the elderly

This study investigated the effects of demographic and built environment factors of urban communities on recreational physical activity among the elderly through multivariate linear regression models. Leisure physical activities among the urban elderly are taken as a dependent variable using the demographic factors (average monthly income, chronic disease, sex, age, highest level of education, living situation, and self-evaluated health) as the first layer of the model and built environment factors (traffic safety, street pavement slope, street connectivity, access to exercise facilities, access to service facilities, residential density, geographic location, cultural landscape, natural landscape, and community safety) as the second layer of the model to investigate the effects of demographic characteristics and built environment factors on recreational physical activity among the elderly.

Model 1 (see Table 5) shows that the sociodemographic variable average monthly income had significant associations with recreational physical activity. Compared with those earning below 1000 ¥ per month, the recreational physical activity of the elderly with average monthly incomes of 1001 to 2000 ¥ (β = 23.31, *p* < 0.001) and 2001 ¥ or more (β = 21.15, p < 0.001) is significantly higher. These results show high average monthly income can promote recreational physical activity in elderly people. Each additional unit of average monthly income can increase recreational physical activity by 23.31 and 21.15 units, respectively. There was an absence of a statistically significant correlation between chronic disease, sex, age, highest level of education, living situation, self-evaluated health, and recreational physical activity. Model 2 shows that the built environment variables geographic location (β = 6.73, *p* = 0.01), street pavement slope (β = − 6.95, *p* = 0.04), and street connectivity (β = 9.32, p = 0.01) had statistically significant associations with recreational physical activity. After controlling for sociodemographic variables in Model 3, street connectivity (β = 7.34, *p* = 0.03) and street pavement slope (β = − 7.72, *p* = 0.02) were still found to be important built environment factors affecting recreational physical activity among the elderly. Ranked in decreasing order of importance, the influencing factors are street pavement slope (β = − 7.72, *p* = 0.020) and street connectivity (β = 7.34, *p* = 0.030). These factors show that high street connectivity and low street pavement slope can promote recreational physical activity among the elderly. Each additional unit of street connectivity and each unit decrease of street pavement slope can increase physical activity by 7.34 and 7.72 units, respectively.

**Table 5:** Multiple linear regression models affecting factors of leisure physical activity in the elderly

variable	Model 1			Model 2			Model 3		
	β	SE	Sig	β	SE	Sig	β	SE	Sig
Female	Reference						Reference		
Male	5.37	5.06	0.29				6.55	5.05	0.20
60–65 years	Reference						Reference		
66–70 years	−1.72	5.10	0.74				−0.95	5.12	0.85
71–75 years	−3.89	5.75	0.50				−2.85	5.82	0.62
76 or more	−6.73	5.96	0.26				−4.84	6.09	0.43
No disease	Reference						Reference		
Disease	1.00	4.10	0.81				−0.55	4.15	0.89
Below 1000¥	Reference						Reference		
1001–2000¥	23.31	7.03	0.00				20.27	7.10	0.01
2001¥ or more	21.15	5.50	0.00				20.40	5.58	0.00
Living with	Reference						Reference		
Alone	2.55	3.99	0.52				2.33	4.01	0.56
Health poor	Reference						Reference		
Average	8.56	8.02	0.29				9.26	8.01	0.25
Good	6.64	8.01	0.41				6.76	8.05	0.40
Elementary school	Reference						Reference		
Junior high school	7.33	5.45	0.18				3.03	5.69	0.59
Senior high school	10.80	6.06	0.08				5.69	6.36	0.37
University	2.53	8.02	0.75				−1.75	8.17	0.83
Access to fitness				3.63	2.90	0.21	2.54	2.87	0.38
Access to services				−0.84	2.70	0.75	0.28	2.69	0.92
Community security				4.64	2.99	0.12	0.31	3.08	0.92
Cultural attractions				1.39	2.91	0.63	2.15	2.90	0.46
Natural landscape				−3.43	3.20	0.28	−2.74	3.22	0.40
Residential density				0.93	2.71	0.73	0.39	2.68	0.89
Geographical location				6.73	2.55	0.01	4.52	2.57	0.08
Street pavement slope				−6.95	3.4	0.04	−7.72	3.36	0.02
Traffic safety				2.59	3.57	0.47	2.69	3.63	0.46
Street connectivity				9.32	3.31	0.01	7.34	3.36	0.03
Model fit	R^2^ = 0.12	F = 3.96		R^2^ = 0.09	F = 3.66		R^2^ = 0.16	F = 3.03	
Significant	p<0.001			p<0.001			p<0.001		

Dependent variable: total score of physical activity; β is the regression coefficient; SE is the standard error

## Discussion

This study provides some support for using the socio-ecological theoretical model to explain the effects of the built environment and demographic factors on physical activity among the elderly. The demographic factors of average monthly income as well as the built environment factors of street connectivity and street pavement slope affect recreational physical activity that elderly people in Nanjing engage in. Creating a supportive environment (e.g., neighborhoods with high levels of street connectivity and smoothly paved, orderly streets) and improving living standards have positive effects on improving physical activity among the elderly.

Whether elderly people engage in leisure time physical activity is influenced by many demographic factors (e.g., age, sex, highest educational level, average monthly income, living situation, health self-assessment). Some of these factors (e.g., highest educational level, average monthly income) have been found by previous studies to be positively associated with physical activity [29]. The regression analysis in the present study also found that average monthly income has a positive impact on recreational physical activity among the elderly; thus, the higher the monthly average income, the higher the level of leisure-time physical activity. A high monthly average income was an important factors in attracting sedentary older people to initiate leisure-time physical activity. This is consistent with the finding by Yang et al. that elderly people with high incomes are more active in terms of recreational physical activity than elderly people with low incomes (AOR = 1.92, 95% CI: 1.55–2.39) [30]. A possible reason is that recreational physical activity depends primarily on discretionary time. Compared to high-income elderly people, low-income elderly people have relatively less discretionary time, resulting in less recreational physical activity [31]. Higher income is associated with higher education, and people with higher education are more likely to recognize the health benefits of regular physical activity, especially walking. High incomes can also lead to increased shopping and leisure excursions, which in turn increase recreational physical activity [32].

In addition to the average monthly income factor, the results of the present study indicate that age is negatively correlated with recreational physical activity among the elderly, indicating that recreational physical activity tends to decline with age. This result is consistent with a study by Lachman et al. that showed that the proportion of individuals engaging in physical activity decreases with age [33]. This may be due to a decline in muscle strength and mass in elderly people; in fact, after the age of 50, muscle strength decreases by 12.000 to 15.000% every decade [34]. Therefore, elderly people often feel their muscles are too weak to support their movement, which leads to an unwillingness to engage in medium- to high-intensity physical activity. On the other hand, the incidence of chronic illness increases with age. Elderly people usually have two or more types of chronic diseases [34]. Due to medical care provided by family members, including overly protective care (e.g., keeping an elderly father bedridden for fear he would injure himself), elderly people tend to be wary of muscle injury (OR = 0.514), falls, and exacerbating illness, leading to decreased recreational physical activity with increasing age [35]. Additionally, the regression analysis in the present study did not find a causal relationship between gender, whether one lives alone, self-evaluated health, highest level of education, or chronic disease and the level of recreational physical activity engaged in by elderly people (*p* > 0.050).

Participation in recreational physical activity by older people is affected by various factors of the built environment. The present study used a one-on-one, face-to-face approach to investigate the factors of the built environment that influence recreational physical activity among the elderly in Nanjing. Regression analysis showed that street connectivity was positively correlated with recreational physical activity among the elderly (*p* = 0.030). As street connectivity increased, residents spent more time engaging in light physical activity, meaning that greater street connectivity provides community residents with alternative transportation routes to promote walking. The results of this study are consistent with those by Sa et al., who found that high street connectivity is conducive to promoting recreational physical activity among the elderly (OR = 1.65, 95% CI: 1.11–2.46) [36]. However, it differs from the study by Marcucci et al., who believe that higher connectivity indices in the areas where elderly people live may reduce their recreational physical activity [37]. A possible reason for their finding is that non-Chinese elderly persons often walk on streets, and a high street connectivity index results in both a high incidence of traffic accidents and a high mortality rate of the elderly in traffic accidents. In addition, pedestrian deaths caused by motor vehicle accidents have been identified as an important cause of non-natural deaths among the elderly [38]. This not only threatens the safety of elderly persons but also reduces their level of recreational physical activity. In China, recreational and physical activity facilities for the elderly are primarily squares and parks near their community. In addition, traffic flow on roads is relatively less in China, and traffic safety on roads is generally good [39]. Therefore, good street connectivity is conducive to promoting recreational physical activity.

The present study found that street pavement slope affects recreational physical activity among the elderly and is correlated with recreational physical activity (*p* = 0.020) [40]. The results of the present study are consistent with those of a study by Gomez et al. that found a negative correlation between street pavement slope and recreational physical activity among the elderly. Elderly people who lived on streets with a slope of > 5% spent less time walking than those who lived on streets with a slope of ≤5% (POR = 0.6, 95% CI: 0.38–0.97) [40]. The reason may be that elderly people generally move slowly and have reduced bodily sensation and balance; moreover, older adults are often particularly sensitive to physical barriers in their neighborhood environment, which may hinder their engagement in outdoor walking, therefore resulting in a “panic” mentality that discourages leisure time physical activity [41]. Another reason may be that the construction of street pavement fails to achieve strict separation of pedestrians and vehicles (there are no separate bike paths or sidewalks in Nanjing), whereas the traffic volume of bicycles and scooters is high; both factors convince older people that walking on the street pavement is unsafe, further resulting in an overall decline in the level of recreational physical activity [42].

Traffic safety, access to exercise facilities, access to service facilities, residential density, geographic location, cultural landscape, natural landscape, and community safety were not associated with recreational physical activity among the elderly, indicating there is no correlation between the built environment factors mentioned above and recreational physical activity among the elderly in this study (*p* > 0.050). Although a study from Australia showed that easy access to exercise and service facilities is directly proportional to total walking time in elderly people [43], a study by Xin et al. showed that residential density has a strong negative correlation with obesity in China [42]. Australia has made a number of recommendations for land use planning and policy measures that include ensuring that housing for the elderly is constructed within 1 km of facilities and activities, and a safety system has been established for elderly pedestrians that includes a safer road environment, safer vehicles, and lower speed limits. However, China has not proposed any relevant plans or policies, which may explain the difference between Chinese and Australian studies [44]. At the same time, these studies also show that not all built environment factors independently affect recreational physical activity among the elderly. Moreover, various environmental factors may interact due to similar spatial and temporal distribution characteristics. The built environment (e.g., neighborhood with fitness facilities, residential density, greenspaces, public health conditions, and paved streets) will work together to promote positive aging.

Overall, the results related to environmental factors were somewhat surprising, as it was anticipated that the selected neighborhood design variables would have a positive effect on physical activity levels of elderly adults. This study is not without limitations. First, this was a cross-sectional study with a design that may not be ideal for maximizing the accuracy of study variables. Although multiple measures were used to control covariates to minimize potential problems associated with recall bias, some answers may still be unreliable because the survey asked elderly participants to recall past behavior and their degree of satisfaction with the activity environment. Though longitudinal designs and quasi-experimental studies can help to resolve causal relationship problems, they are hard to implement in reality. Second, this study used subjective measurement methods of physical activity (PASE) and built environment (modified NEWS) for elderly people in urban Chinese communities; therefore, the subjects might not have objectively evaluated the impact of built environmental factors on their physical activity. In the future, objective measures (e.g., using higher-quality and more detailed GIS and GPS to measure built environment data and using acceleration GT3X to measure physical activity level) will be used to investigate the correlation between physical activity of elderly people and the built environment in which they live.

## Conclusions

In summary, this study shows that older people with a higher average monthly income are more likely to participate in recreational sports than older people with lower average monthly income. In order to positively impact physical activity in older adults and ultimately improve health, policymakers and urban planners need to ensure that street connectivity and street pavement slope are factored into the design and development of urban environment.

## Data Availability

The datasets generated and/or analyzed during the current study are not publicly available due to maintain participant privacy and confidentiality requirements but are available from the corresponding author on reasonable request.

## Abbreviations

NEWS: Neighborhood environment walkability scale
PASE: Physical activity scale for the elderly
WHO: World Health Organization

## Footnotes

1
http://www.drjamessallis.sdsu.edu/measures.html
